# Multi-scale pore characterization of vitrain and durain in low rank coal

**DOI:** 10.1038/s41598-025-19868-w

**Published:** 2025-10-14

**Authors:** Chao Zheng, Yue Chen, Wulin Lei, Jufeng Zhang

**Affiliations:** 1 College of New Energy, Long Dong University, Qingyang, 745000 China; 2 College of Geology and Environment, Xi’an University of Science and Technology, Xi’an, 710054, China

**Keywords:** Vitrain, Durain, Super-micropore, Full pore size distribution, Effective porosity, Energy science and technology, Engineering, Solid Earth sciences

## Abstract

Coal is composed of multiple macroscopic compositions, and its complex pore-fracture system determines the adsorption and desorption capability of coalbed methane (CBM). In this paper, the multi-scale method were used to finely characterize the full pore size distribution of vitrain and durain, the differences of micropore and effective porosity between them were emphatically analyzed. The results indicated that the pore specific surface area (SSA) and pore volume (PV) of vitrain exceed those of durain via full pore size distribution analysis, primarily attributable to the influence of the super-micropore (0.6 nm ~ 0.85 nm). For pore characteristics affecting methane diffusion and seepage, the effective porosity ratio ranges from 9.8% to 35.1%, all of which are less than 50%, reflecting that much pores in coal reservoirs are closed pores. The effective porosity, full-scale average pore size and pore connectivity of durain are all superior to those of the corresponding coal samples of vitrain. These characteristics indicate that the pores of durain are more conducive to fluid migration. This provides a profound understanding for the efficient development of CBM in low-rank coal.

## Introduction

Coal reservoirs are naturally complex heterogeneous porous media, the pore-fracture system within them provides space and channels for the enrichment, storage and migration of coalbed methane (CBM)^1–5^. The pore characteristics of coal reservoirs are directly related to the adsorption/desorption capacity, diffusion and permeability of methane. Coal matrix pores (especially micropores) are the storage space for methane, fractures are the channels for the migration, and micro-fractures are the bridges between pores and fractures^6–9^. Therefore, the pore size distribution characteristics and connectivity (diffusion/permeability) ultimately become the key factors restricting the gas content and productivity^10,11^. Comprehensive and fine analysis of pore characteristics is basic research for CBM resource evaluation and large-scale development.

The research on the pore characteristics of coal reservoirs generally includes the classification and genesis types, the influencing factors of pore development, and the fine characterization methods of pore structure^12^. In recent years, new technologies such as NMR^13–15^ X-ray CT^16–18^, AMF^19^ FIB-SEM^20^ and SAXS^21^ etc. and multifractal theory^22–25^ have been applied to the study of pores-fractures, significantly improving the characterization accuracy of nanoscale micropores in coal reservoirs^26,27^. In terms of the pore characteristics, the degree of coal metamorphism affects the methane adsorption/desorption capacity by influencing the pores development. As the coal rank increases, pore volume (PV) and specific surface area (SSA) of micropores increase, while PV and SSA of transitional pores and mesopores decrease^28,29^ i.e. the higher the degree of metamorphism, the better the pore development. The outburst coal having the larger PV and SSA and the more complex microstructure^30^, tectonic stress has the dual effect on the transformation between closed pores and open pores of coal in the process of deformation^31^. Pore structure characteristics has obvious impact on gas desorption of coking coal. Positive correlations have been recognized between gas desorption capacity and PV, SSA and the average pore size^32^. The SSA, PV and fractal dimension associated with micropores in coal showed a trend of first decrease and then increase with increase in coal rank^33^. The SSA and PV of micropores in low-rank vitrain are higher than those in durain, while the SSA and PV of mesopores and macropores are lower than those in durain^34^. Vitrain is characterized by cleats, micropores and microfractures which are often filled up partially or completely with mineral matter^35^. Liu et al^21^. studied the characteristics of open pores and closed pores in different coal rank. Influenced by the molecular structure, the proportion of closed pores increased simultaneously as the coal rank increased. In terms of the number of pores, the number of closed pores is 1 ~ 3 orders greater than that of open pores. The formation of closed pores is partly because of the collapse of the internal structure and partly because of the volatilization of unstable substances^36^. In conclusion, the study of the pore characteristics of coal reservoirs is the traditional research topic and has also got the wealth of research results. However, at present, the research on the pore characteristics of the macroscopic compositions, especially the micropore & effective pore characteristics that restrict methane adsorption and desorption, is still insufficient^37–41^. In this context, the fine characterization of coal reservoirs is the important approach to revealing the pore development characteristics of the reservoirs.

The pores in coal reservoirs cover multiple-scales ranging from micropores to fractures, and a single analytical method is bound to have certain errors. This study employed a full-scale characterization method covering micropores, mesopores, macropores, and fractures to comprehensively analyze, the key pore characteristics that affect methane adsorption/desorption, particularly highlighted the differences between vitrain and durain in terms of micropore distribution, average pore size, effective porosity, and pore connectivity. The study aims to deepen the theory of CBM development.

### Sample and methods

**Fig. 1:**
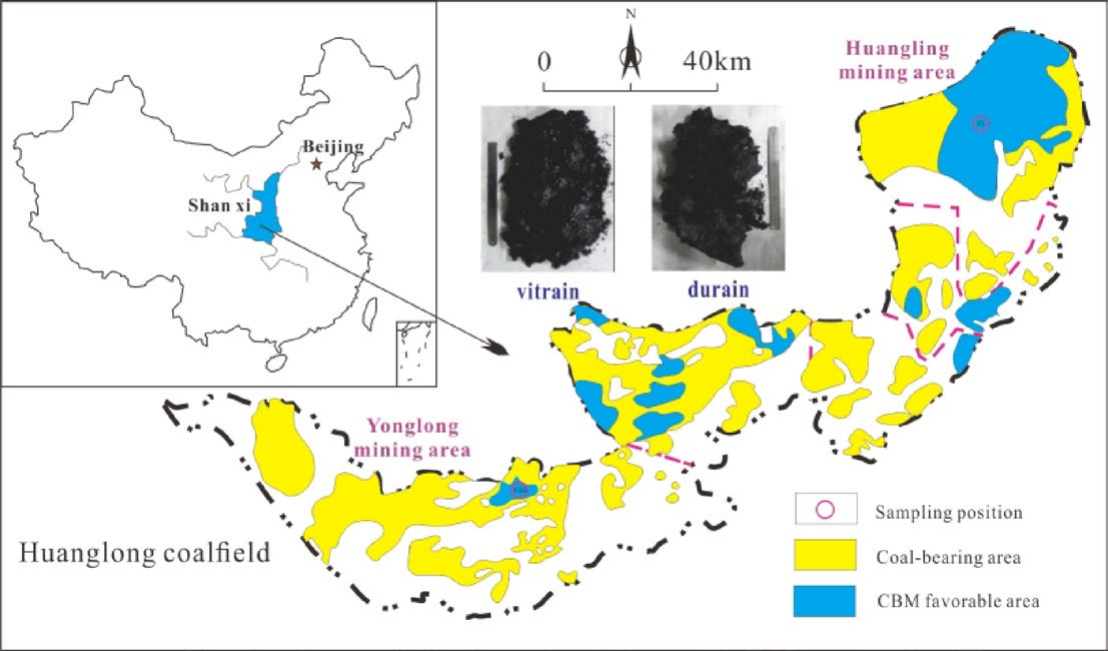
Location of coal sample collection. Modified from^37^.

The coal samples in this study originate from two low-rank coal mine in the Huanglong coalfield, located on the southwestern margin of the Ordos Basin (Fig. 1). The samples are designated as YZG coal (*R*_o, max_=0.65%) and HL coal (*R*_o, max_=0.68%). To compare and analyze the full pore characteristics of both vitrain (YZG-JM, HL-JM) and durain (YZG-AM, HL-AM). According to the differences in color and luster, the columnar samples for low field nuclear magnetic resonance (LF-NMR) analysis are segmented from the vitrain strips (Dark black & strong luster) of the large coal samples using wire-cutting technology, the mercury intrusion porosimetry (MIP) etc. test samples are separated by hand.

**Fig. 2:**
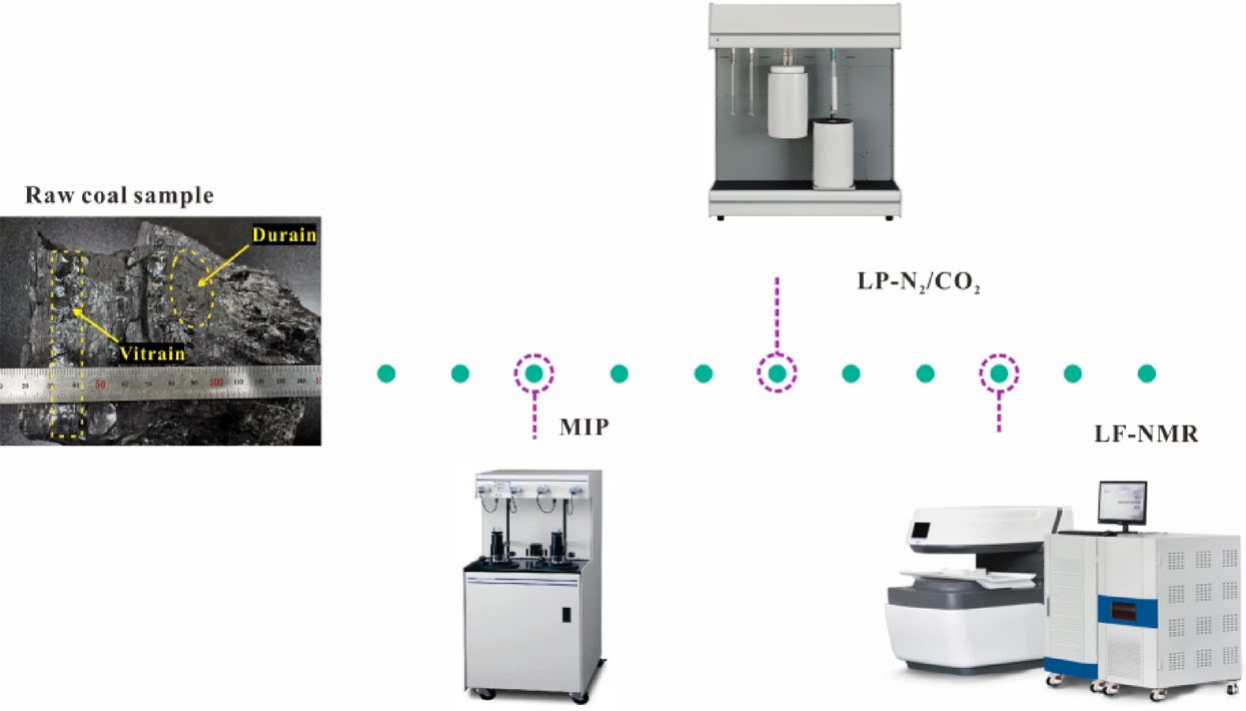
The experimental equipment in the research.

This study employed multi-scale methods with different testing principles to analyze the pore characteristics of vitrain and durain. These methods comprehensively covered the entire scale of pores from micropores, macropores to fractures. Among them, the fluid intrusion method is the MIP, and the gas adsorption method includes LP-N_2_ adsorption and LP-CO_2_ adsorption, LF-NMR is a non-destructive testing method. Moreover, the results of LF-NMR can mutually verify the results obtained by methods such as MIP, LP-N_2_, LP-CO_2_, etc. The MIP can be used to analyze macropore pores in coal reservoirs with pore sizes ranging from 0.005 μm to 1000 μm, and the sample size is 1.0 cm×1.0 cm×1.0 cm small cubic samples. LP-N_2_/LP-CO_2_ are mainly used for testing mesopore and micropores in coal reservoirs, the pore size of LP-N_2_ adsorption can be analyzed in the range of 2 ~ 300 nm, while CO_2_ molecules are smaller, and the pore size can be analyzed in the range of 0.2 ~ 1.6 nm. LF-NMR was used to analyze pore proportion, separability and continuity by comparing pressurized saturated water and centrifugally bound water samples with positively charged hydrogen core (^1^H) fluid as probe. The size were cut Φ2.5 cm×5.0 cm columnar samples. The experimental equipment is shown in Fig. 2.

## Results and dicussion

### Pore development characteristics via MIP

**Fig. 3:**
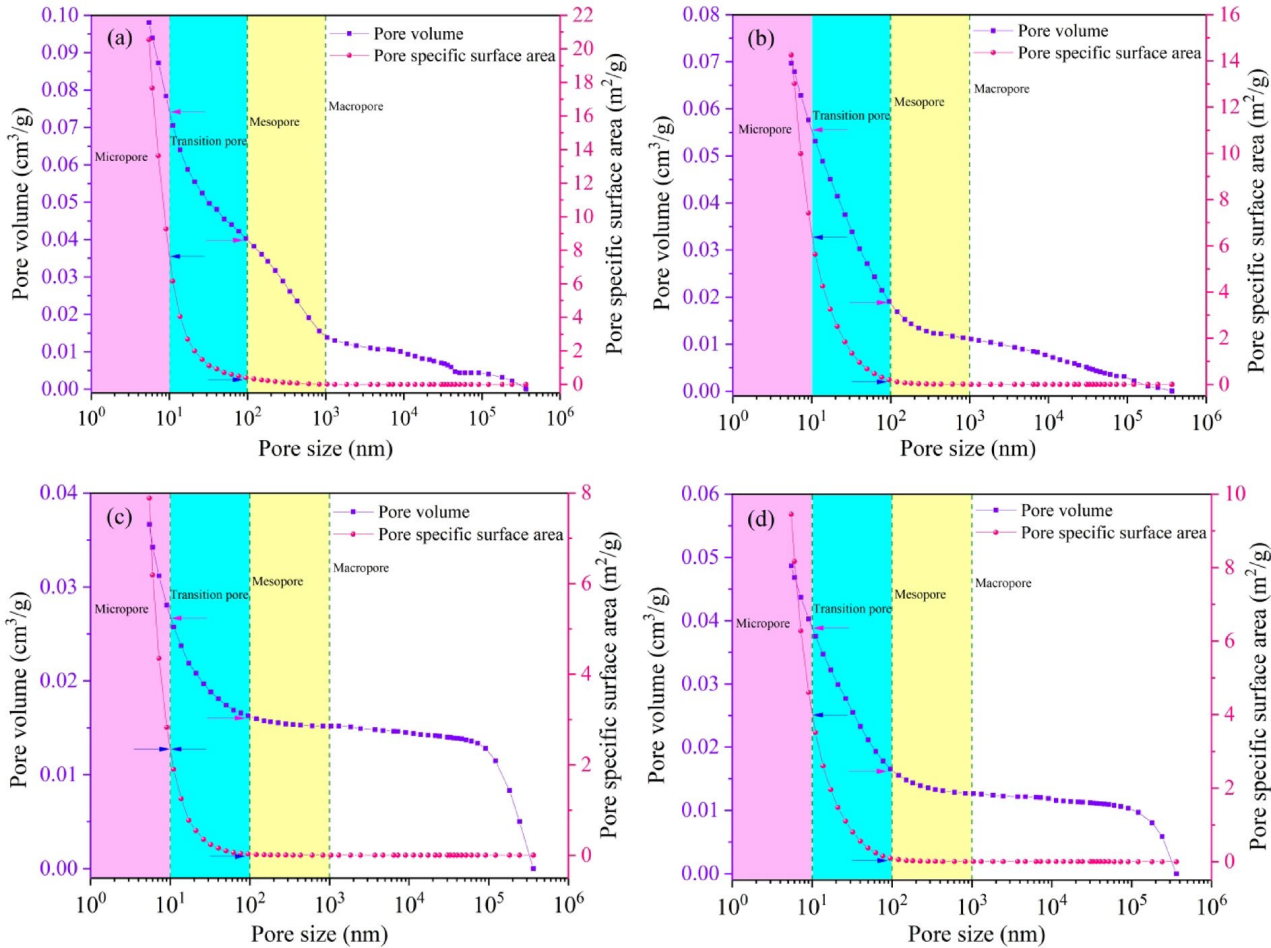
Pore size distribution **(a)** YZG-JM, **(b)** YZG-AM,**(c)** HL-JM, **(d)** HL-AM.

According to the B.B.Ходот pore size classification standard, micropores (< 10 nm), transitional pores (10 ~ 100 nm), mesopores (100 ~ 1000 nm), and macropores (> 1000 nm), the MIP analysis results of vitrain and durain are shown in Fig. 3. The pore volume (PV) and specific surface area (SSA) exhibit different variation characteristics at different pore size scope. That is, within different pore size, the growth rates of PV and SSA are different, and the contributions to the PV and SSA are significantly different. In terms of PV, the comparative analysis of YZG-JM and YZG-AM shows that at the macropore rang, the PV is 0.15 cm^3^/g for both, the growth of mesopores and transitional pores in YZG-JM is basically consistent, but the contribution of mesopores in YZG-AM is smaller, and the growth of transitional pores is significant. For the HL coal sample, the cumulative PV in the macropore and mesopore is 0.15 cm^3^/g, but the growth of HL-AM within the transitional pore scope is significantly greater than that of HL-JM. In terms of SSA, the four coal samples have almost no contribution in the macropore, and YZG-JM and YZG-AM differ little. In the transitional pore stage, HL-JM has a slower growth rate than HL-AM.

The analysis results of the MIP are shown in Table 1. Except for HL-JM, the transitional pores are the main contributors to the PV; while the micropores are the main contributors to the SSA, accounting for more than 60%. In the same coal sample, the PV of vitrain is greater than that of durain, and there is a difference in the pore SSA. The possible reason is that the MIP primarily targets macropores and fissures, and its testing of micropores is not precise enough to characterize their influence.

**Table 1:** PV and SSA distribution of vitrain and durain with MIP.

Coal sample	PV/Distribution					SSA/Distribution				
	PV (m^3^/g)	Micropore (%)	Transition pore (%)	Mesopore (%)	Macropore (%)	SSA (m^2^/g)	Micropore (%)	Transition pore (%)	Mesopore (%)	Macropore (%)
YZG-JM	0.098	28.13	32.93	24.87	14.07	20.54	70.03	28.35	1.58	0.04
YZG-AM	0.069	23.82	51.94	8.32	15.93	14.26	60.54	38.63	0.79	0.04
HL-JM	0.036	29.70	26.70	2.18	41.42	7.89	75.92	23.86	0.19	0.02
HL-AM	0.048	23.00	44.97	5.95	26.08	9.45	62.85	36.55	0.58	0.02

### Pore development characteristics via LP-N_2_ adsorption

**Fig. 4:**
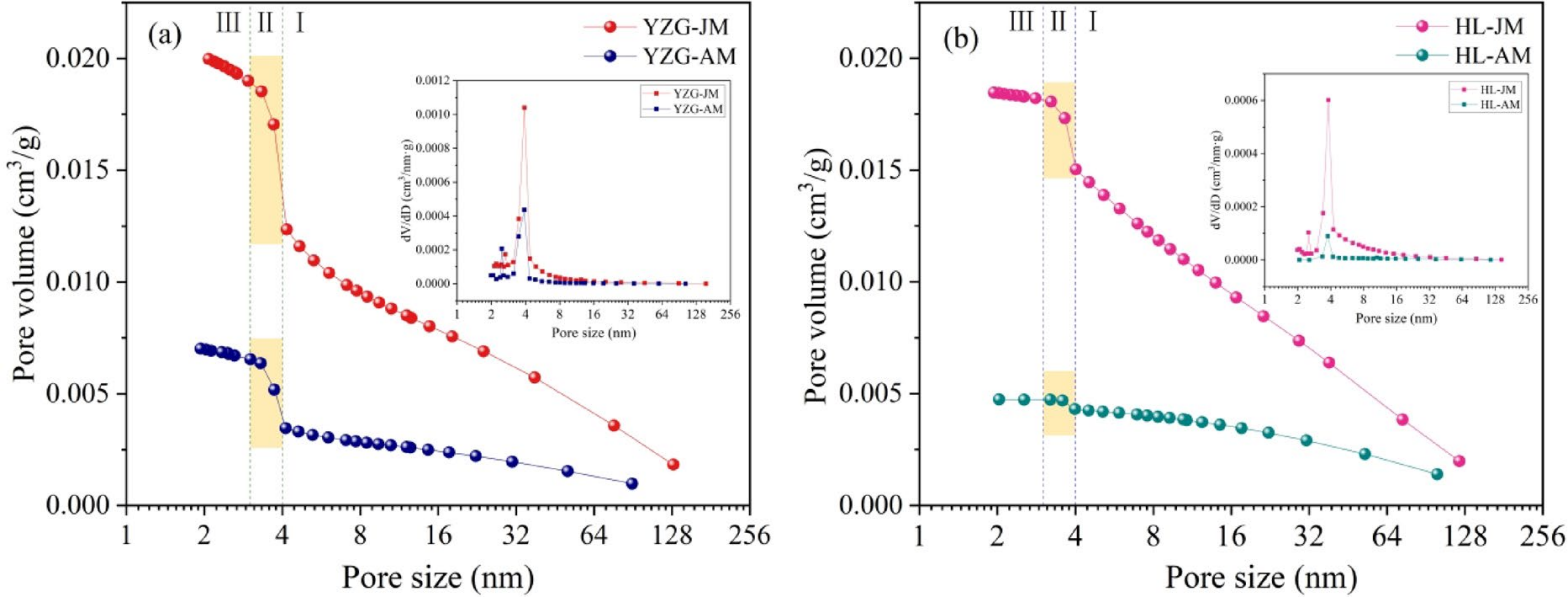
PV distribution with LP-N_2_ adsorption **(a)** YZG, **(b)** HL.

**Fig. 5:**
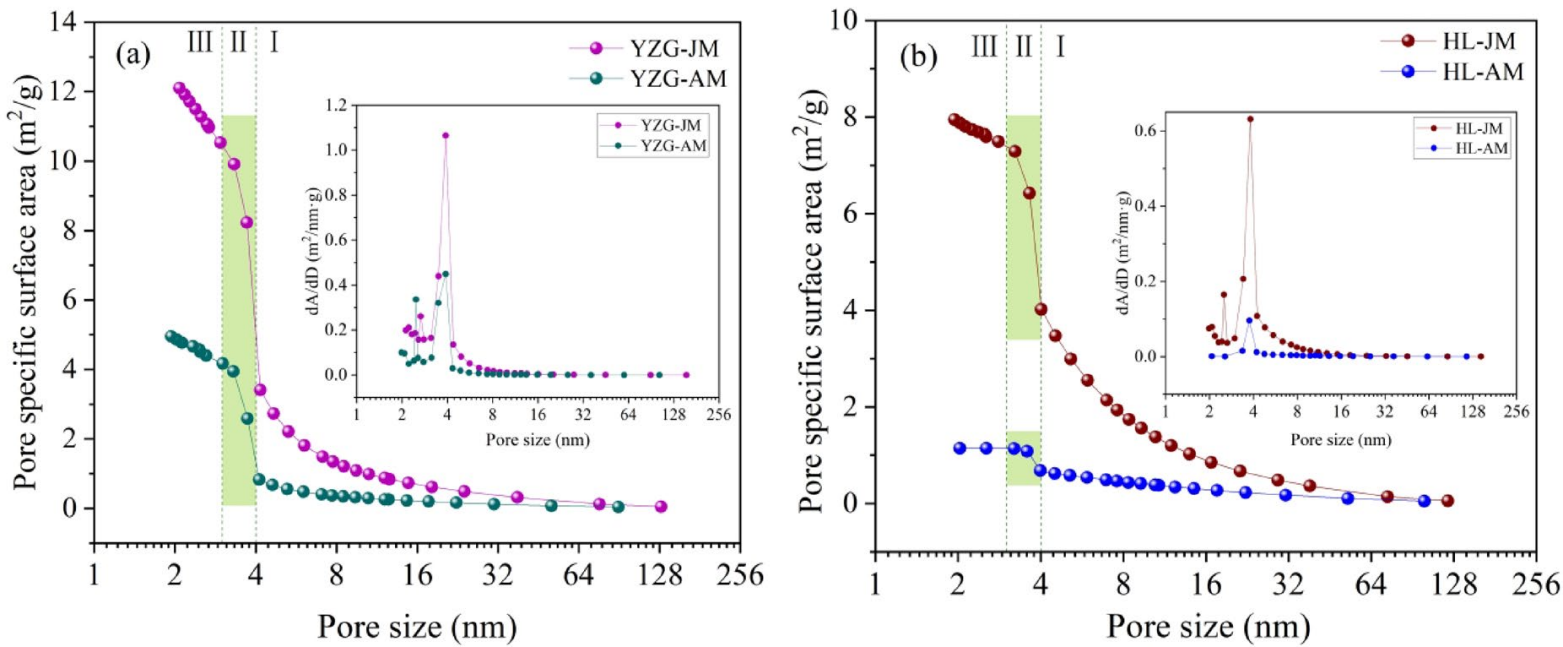
Pore SSA distribution with LP-N_2_ adsorption **(a)** YZG, **(b)** HL.

According to the LP-N_2_ adsorption results, the PV and SSA of vitrain are greater than durain within the pore size 1.8 ~ 145 nm. However, based on the slope of the curve, the PV and SSA curves can be divided into three distinct stages (Figs. 4 and 5). In the stage Ⅰ, the PV and SSA increase slowly, the PV curve can be approximated as a linear function with pore size 4~ 145 nm, and this stage contributes the most to the PV; while the SSA curve is an exponential function, indicating that the growth rate of the SSA variation with the pore size; i.e. as the pore size decreases, the SSA increases significantly. In the stage Ⅱ, with pore size 3 ~ 4 nm, the PV and SSA increase significantly, and this stage contributes the most to the SSA. In the stage Ⅲ, with pore sizes 1.8 ~ 3 nm, the slope of the curve decreases again and increases slowly. Comparative analysis reveals the contribution of the stage Ⅲ to both the PV and SSA is not significant. The reason for this is that the micropores have a relatively small contribution to PV with pore size 1.8 ~ 3 nm; for the SSA, due to the technical limitations of LP-N_2_ adsorption, the micropore characterization is not ideal.

Based on the LP-N_2_ adsorption results, through the analysis and calculation using the BET and BJH models, the pore SSA of YZG-JM is 2 ~ 3 times that of YZG-AM, and the SSA of HL-JM is 5 ~ 7 times that of durain. In terms of the average pore size, the pores size of durain are larger than those of vitrain (Table 2).

**Table 2:** Pore characteristics of vitrain and durain with LP-N_2_ adsorption.

Coal sample	BET		BJH		
	SSA (m^2^/g)	Average pore size (nm)	SSA(m^2^/g)	Average pore size (nm)	PV (cm^3^/g)
YZG-JM	10.606	5.531	12.295	6.513	0.0201
YZG-AM	4.756	6.615	4.952	5.668	0.0071
HL-JM	6.894	9.271	8.008	9.240	0.0185
HL-AM	1.269	12.458	1.144	16.558	0.0047

### Pore development characteristics via LP-CO_2_ adsorption

Due to its test limit for pores size > 1.8 nm, LP-N₂ adsorption provides inaccurate micropore characterization. In contrast, CO₂ molecules are smaller than N₂, and can access narrower pores, making LP-CO₂ adsorption/desorption analyzed via the DFT model the more objective method for characterizing micropores (< 2 nm). As shown in Table 3, vitrain consistently exhibits larger SSA than durain across both models (variation: 6.25–15.8%). Conversely, BET measurements indicate larger average micropore size in durain, with values of 0.975 nm (YZG-JM), 1.018 nm (YZG-AM), 0.752 nm (HL-JM), and 1.023 nm (HL-AM).

**Table 3:** Pore characteristics of vitrain and durain with LP-CO_2_ adsorption.

Coal sample	BET micropore SSA (m^2^/g)	BET average pore size (nm)	DFT micropore SSA (m^2^/g)			DFT micropore volume (cm^3^/g)	
			> 0.384 (nm)	> 1.066 (nm)	0.384 ~ 1.066 (nm)	< 1.066 (nm)	< 0.384 (nm)
YZG-JM	150.737	0.975	160.51	75.339	85.171	0.02535	0.00025
YZG-AM	114.937	1.018	127.417	56.338	71.079	0.02097	0.00014
HL-JM	139.559	0.752	117.623	65.943	51.680	0.01503	0.00000
HL-AM	101.589	1.023	113.924	49.502	64.422	0.01866	0.00000

**Fig. 6:**
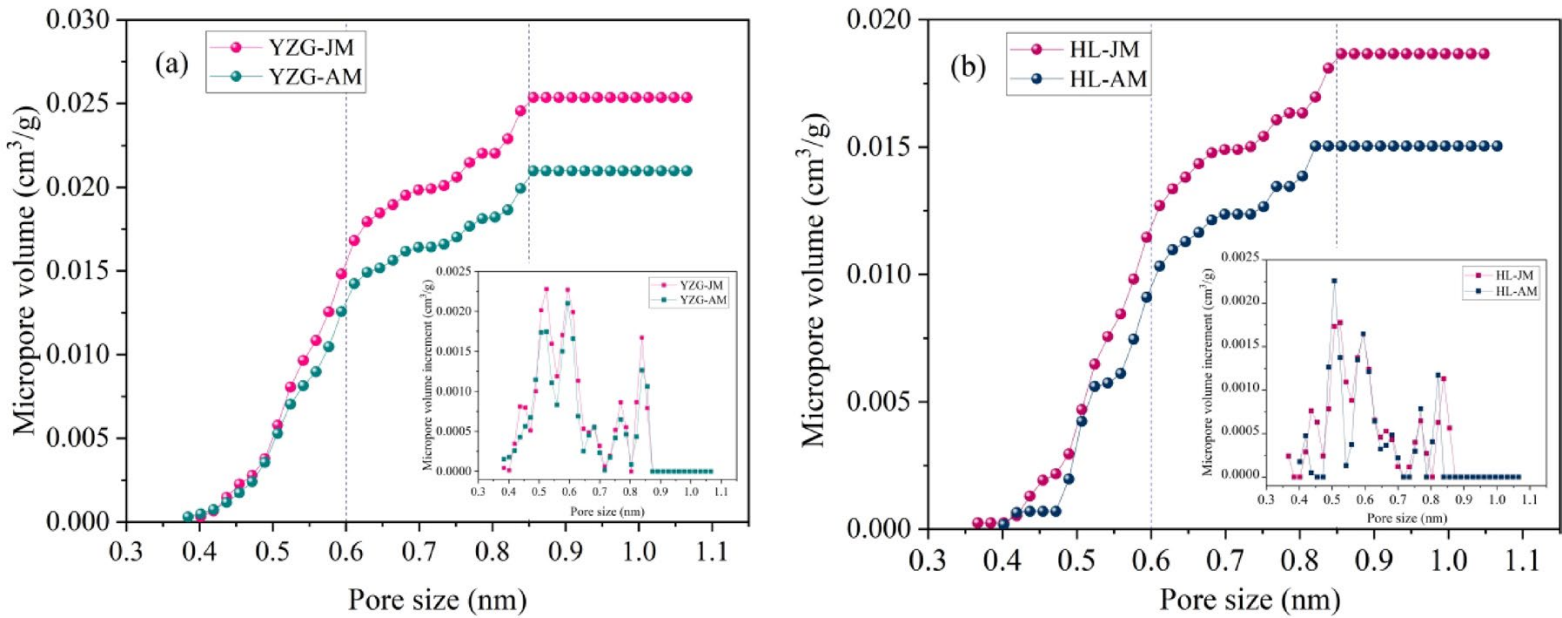
PV distribution by LP-CO_2_ adsorption **(a)** YZG, **(b)** HL.

**Fig. 7:**
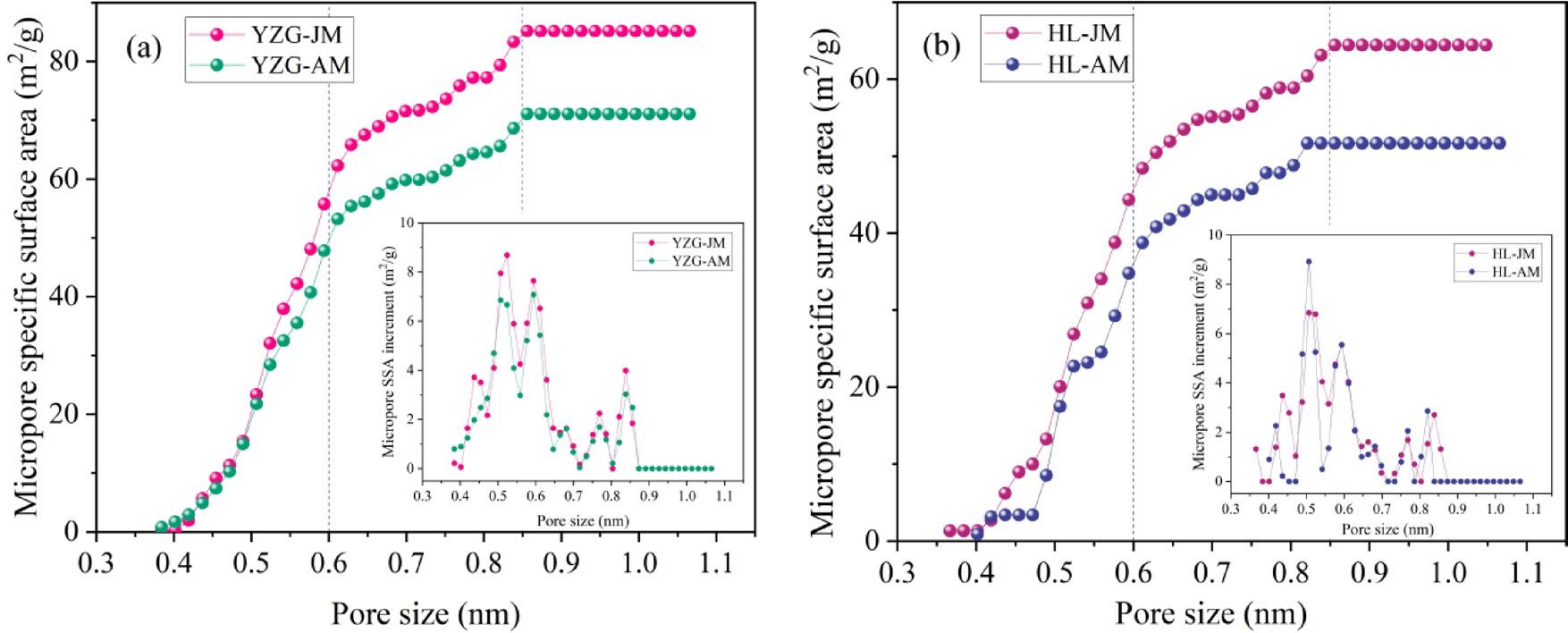
Pore SSA distribution by LP-CO_2_ adsorption **(a)** YZG, **(b)** HL.

According to the characteristic curve between PV and pore size (Fig. 6), and the characteristic curve between pore SSA and pore pore size (Fig. 7). It is found that the change curve between pore size and PV/SSA can be divided into three stages: ultra-micropore (< 0.6 nm), supermicropore (0.6 ~ 0.85 nm) and micropore (> 0.85 nm). At the ultra-micropore size, the curve is steeper, the slope is larger, and the PV and SSA increase obviously, but there is little difference between vitrain and durain. At the super-micropore, the curve is slower and the slope is lower than that of the first stage, but the slope of vitrain curve at this stage is significantly larger than that of durain, and the gap between the PV and SSA of vitrain and durain is significantly wider, and the vitrain curve is above durain. At the micropore size (> 0.85 nm), the curve is almost horizontal and the slope is basically zero. Therefore, it can be considered that the difference in PV and SSA between vitrain and durain is mainly attributed to the super-micropore.

### Full pore scales distribution of vitrain and durain

The pores in coal span many scales in size and are widely distributed from micropores to macropores. Due to the influence of molecular probe, measuting principle and calculation model etc., such as MIP and gas adsorption method can only analyze the pore size at a certain scope, and it is difficult to accurately characterize the full pore size distribution of coal. According to the results of MIP, LP-N_2_ and LP-CO_2_ adsorption, IUPAC pore classification was adopt to characterize full pore scales distribution of vitrain and durain. The PSD of full pore scales of vitrain and durain are shown in Tables 4 and 5. The results show that macropores are still the largest contributor to PV, accounting for more than 50% of the total pore volume (TPV), followed by micropores, with the smallest proportion of mespores. For pore SSA, micropores are the most important contributors, all of which are above 90%, and the rest are mesoporous and macropores in turn. In the same coal sample, the PSD of full pore scales of vitrain and durain are similar to the results of single test. MIP and LP-N_2_ adsorption are not accurate enough to measure micropores. The data of full pore scales micropores are derived from LP-CO_2_ adsorption, so the SSA of pores increases significantly. The total pore volume (TPV) and total specific surface area (TSSA) of vitrain are larger than those of durain in both YZG and HL coal samples. In terms of pore SSA, which plays the decisive role in CBM adsorption, vitrain has obvious advantages in the development of micropores. As shown in Table 6, for the average pore size of vitrain and durain, the results are consistent by different methods (MIP, LP-N_2_, LP-CO_2_), that is, the average pore size of durain is larger than that of vitrain, indicating that at the same pore scale, the pore size of vitrain is small, while that of durain is relatively large.

**Table 4:** PV distribution of full pore scales of vitrain and durain.

Coal sample	TPV (cm^3^/g)	PV (cm^3^/g)			PV proportion (%)		
		V_1_	V_2_	V_3_	V_1_	V_2_	V_3_
YZG-JM	0.07975	0.02535	0.0089	0.0455	31.79	11.16	57.05
YZG-AM	0.05187	0.02097	0.0038	0.0271	40.43	7.33	52.24
HL-JM	0.04073	0.01503	0.0045	0.0212	36.90	11.05	52.05
HL-AM	0.03646	0.01866	0.0004	0.0174	51.18	1.09	47.73

Note: V_1_: Micropore(< 2 nm); V_2_: Mesopore(2 nm ~ 50 nm); V_3_: Macropore(> 50 nm).

**Table 5:** Pore SSA distribution of full pore scales of vitrain and durain.

Coal sample	TSSA (m^2^/g)	SSA (m^2^/g)			SSA proportion (%)		
		S_1_	S_2_	S_3_	S_1_	S_2_	S_3_
YZG-JM	170.919	160.51	9.703	0.7064	93.91	5.68	0.41
YZG-AM	132.302	127.42	4.211	0.6709	96.31	3.18	0.51
HL-JM	122.872	117.62	4.884	0.3683	95.74	3.97	0.29
HL-AM	114.572	113.924	0.561	0.0972	99.43	0.48	0.09

Note: S_1_: Micropore(< 2 nm); S_2_: Mesopore(2 nm ~ 50 nm); S_3_: Macropore(> 50 nm).

**Table 6:** The average pore size based on multi-scale methods.

Coal sample	MIP pore size (nm)	LP-*N*_2_ pore size (nm)	LP-CO_2_ pore size (nm)
YZG-JM	19.10	5.531	0.975
YZG-AM	19.55	6.615	1.018
HL-JM	18.60	9.271	0.752
HL-AM	20.59	12.458	1.023

### Effective porosity and connectivity via NMR

The PSD analysis results by NMR was shown in Table 7. According to the IUPAC pore classification, YZG-JM and HL-JM micropores (< 2 nm) the proportions is 46.99% and 75.52%, respectively; The micropore proportions of YZG-AM and HL-AM is 30.39% and 36.08% respectively, that is, micropore development of vitrain is higher than that of durain, but mesoporous development of durain is higher than that of vitrain. In contrast, the total porosity indicate the number of pores in the coal reservoir, furthermore the effective porosity of the reservoir plays important role in the fluids transport. Effective porosities of YZG sample are 1.76% (YZG-JM) and 2.43% (YZG-AM), durain exhibits 1.38× higher effective porosity than vitrain. Similarly, HL effective porosities (YZG-JM: 0.66%; YZG-AM: 2.34%) show durain/vitrain ratio of 3.55. Meanwhile, the porosity ratio represents the proportion of effective porosity to total porosity, and reflects the proportion of connected pores in the pores. The calculation shows that the porosity ratio of the four samples is less than 50%, indicating that more than 50% of the pores in the coal samples are closed pores, which is not conducive to the desorption and transport of methane. That is, the effective porosity of durain is large, and its permeability is also large, which facilitates the diffusion and seeping of methane.

**Table 7:** NMR pore characteristics of vitrain and durain.

Coal sample	Pore distribution (%)			Effective porosity	Porosity ratio
	Macropore	Mesopore	Micropore		
YZG-JM	9.86	44.18	46.99	1.76%	9.80%
YZG-AM	7.99	61.62	30.39	2.43%	14.5%
HL-JM	10.59	13.89	75.52	0.66%	13.4%
HL-AM	32.78	31.14	36.08	2.34%	35.1%

**Fig. 8:**
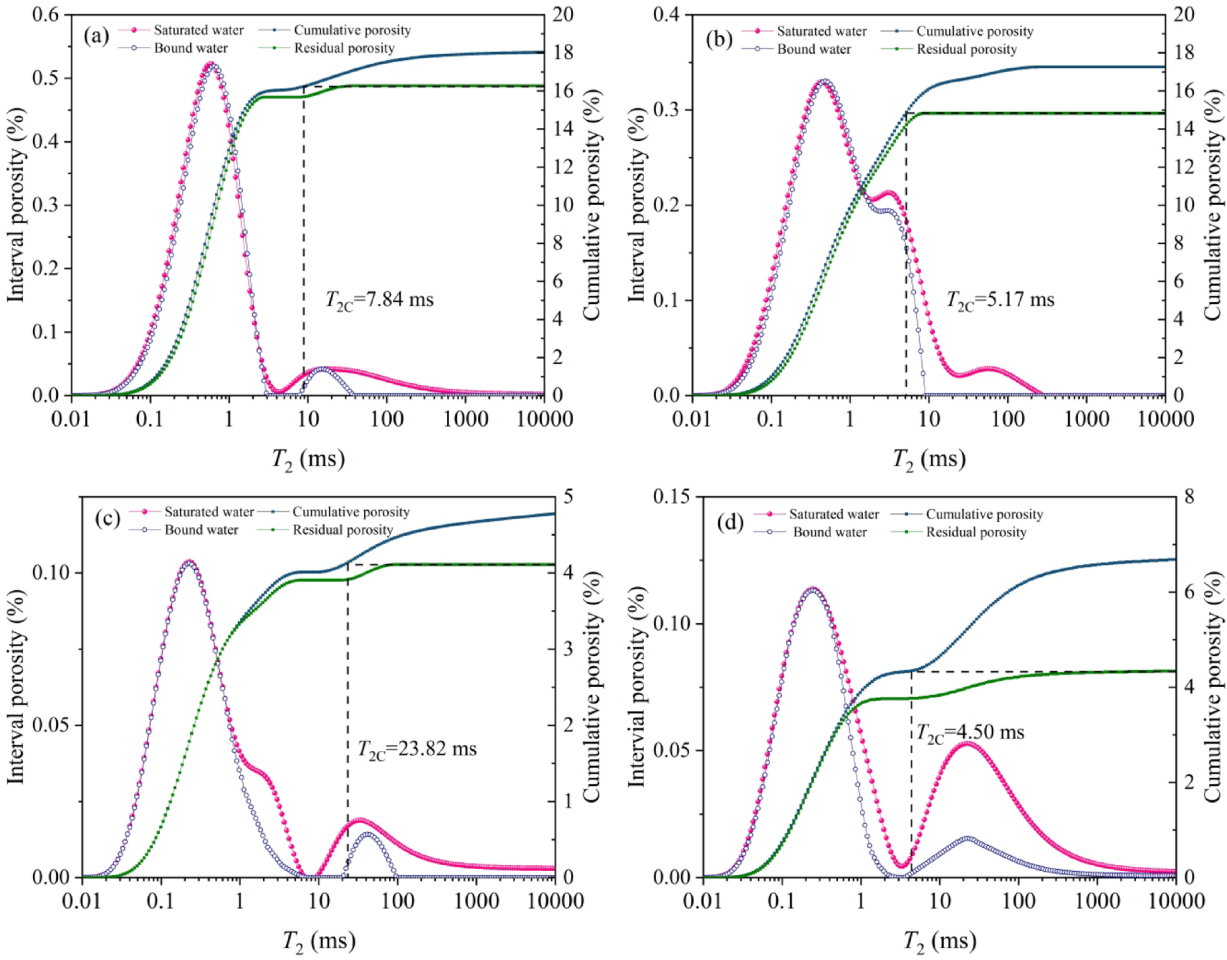
*T*_2c_ spectrum **(a)** YZG-JM, **(b)** YZG-AM, **(c)** HL-JM, **(d)** HL-AM.

According to the NMR analysis principle, the morphology of NMR *T*_2_ spectrum can effectively reflect the proportion of different pores, in which the time in the *T*_2_ spectrum corresponds to different pore size, the area and width of the peak indicate the pore proportion and the sortability of the pores, and the continuity of peaks reflects the connectivity of pore size^42^. The *T*_2_ spectrum of YZG-JM is a bimodal curve, while YZG-AM is a trimodal curve, indicating the development of mesopores within it. The HL coal sample basically presents the bimodal curve (Fig. 8). Compared with YZG-JM and HL-JM, the peak of YZG-AM and HL-AM connect more smoothly, suggesting good connectivity between different-size pores, resulting in significant differences in the area between the bound water and saturated water curves of YZG-AM and HL-AM, reflecting the smooth flow of free water under centrifugal conditions.

Combined with the water-saturated and centrifugation joint measurement method, according to the different relaxation times of various pore-throat in the coal, the *T*_2_ spectrum shows different spectral peak characteristics, and then judges the pore characteristics. When the pore is saturated with water, the occurrence forms of water in pores include two types: free state and bound state, and the bound state is mainly capillary water and thin film water^43^. The *T*_2_ relaxation time corresponding to the bound fluid is short, and the *T*_2_ relaxation time corresponding to the free fluid is long. According to the *T*_2_cutoff value, the reservoir fluid is divided into movable fluid and immovable fluid, and the *T*_2C_ value is the critical value of effective porosity and residual porosity^44^. Through the analysis of the total porosity and *T*_2C_ value of the sample (Fig. 8), the *T*_2C_ values of YZG-JM and YZG-AM are 7.84 ms and 5.17 ms, respectively, and those of HL-JM and HL-AM are 23.82 ms and 4.50 ms, respectively. The durain is smaller than the vitrain and closer to the left of the *T*_2_ horizontal axis. It shows that the water content in vitrain changes relatively little under centrifugation, the proportion of bound water is large, and the effective porosity is low, which is consistent with the direct test results.

The characteristics of micropores in the pores of coal reservoirs are the key factors determining methane adsorption/desorption. The results of full PSD show that TPV and SSA of vitrain are larger than that of durain, in which macropores are still the main contributor to PV, accounting for more than 50% of the TPV; micropore is the largest contributor of pore SSA, all of which are above 90%. This results corroborates the proposition by Lin et al.^45^. It must be noted that the difference in PV and SSA between vitrain and durain is mainly attributed to the super-micropore (0.6 ~ 0.85 nm), meanwhile, Li et al.^33^ found that SSA and PV are controlled mainly by pore diameters of 0.55 ~ 0.6 nm, 0.8 ~ 0.83 nm and 1.2 ~ 1.3 nm in low to high-rank coals. There indicates that super-micropores are of paramount importance across different coal rank and among different macroscopic composition.

The study analyzed the average pore size of vitrain and durain across three scales-micropores, mesopores and macropores. Full pore scales distribution shown that the pore size of vitrain ranges is 0.752 ~ 19.10 nm, and that of durain is 1.018 ~ 20.59 nm, the average pore size of durain is larger than that of vitrain at the same pore scale. Compared with previous investigation^37^ that did not distinguish pore scales, the pore size in this study were measured more comprehensive.

The effective porosity ratio of coal sample ranges from 9.76% to 35.1%, all of which are less than 50%, reflecting that much pores in coal reservoirs are closed pores, the effective porosity of durain are more than that of vitrain. Most previous studies focused on the porosity in coal reservoirs. In fact, the closed pores is pervasive in coal reservoirs, accounting for over 94% of the total pores^36^. Hence, this study not only proved this viewpoint but also revealed the differences in effective porosity between vitrain and durain.

The results of NMR indicate that the *T*_2_ spectral in durain are smoother than those in vitrain, and the *T*_2C_ value of durain is smaller than that of vitrain, which reflects the pore connectivity is better than that in vitrain. Compared with literature^37^ etc., the research conclusion is consistent. However, previous studies mainly conducted qualitative analysis based on the continuity of the *T*_2_ spectral. This study is a qualitative study based on *T*_2C_ value.

## Conclusion

In this study, the multi-scale method were used to finely characterize the full pore size distribution of vitrain and durain, the differences of micropore and effective porosity between them are emphatically analyzed. The main conclusions are as follows.

1. Micropores and fractures of vitrain development, while mesoporous development of durain, TPV and SSA of vitrain are larger than that of durain. The difference in PV and SSA between vitrain and durain is mainly attributed to the super-micropore (0.6 ~ 0.85 nm).
2. The effective porosity ratio of coal sample ranges from 9.76% to 35.1%, all of which are less than 50%, reflecting that much pores in coal reservoirs are closed pores, the effective porosity of durain are more than that of vitrain. The pore size of vitrain ranges is 0.752 ~ 19.10 nm, and that of durain is 1.018 ~ 20.59 nm, i.g. the average pore size of durain is greater than that of vitrain across all scales.
3. The *T*_2_ spectral in durain are smoother than those in vitrain. Moreover, the *T*_2C_ value of durain is smaller than that of vitrain, which reflects the pore connectivity is better than that in vitrain.
4. The study have deepened our understanding of the pore characteristics of vitrain and durain in low-rank coal. In the future, advanced methods should be employed to separate vitrain from durain, and studies on the macroscopic compositions of different coal-rank should be conducted.

## Data Availability

All data generated or analysed during this study are included in this published article.
